# Toward a “treadmill test” for cognition: Improved prediction of general cognitive ability from the task activated brain

**DOI:** 10.1002/hbm.25007

**Published:** 2020-05-04

**Authors:** Chandra Sripada, Mike Angstadt, Saige Rutherford, Aman Taxali, Kerby Shedden

**Affiliations:** ^1^ Department of Psychiatry University of Michigan Ann Arbor Michigan USA; ^2^ Department of Statistics University of Michigan Ann Arbor Michigan USA

## Abstract

General cognitive ability (GCA) refers to a trait‐like ability that contributes to performance across diverse cognitive tasks. Identifying brain‐based markers of GCA has been a longstanding goal of cognitive and clinical neuroscience. Recently, predictive modeling methods have emerged that build whole‐brain, distributed neural signatures for phenotypes of interest. In this study, we employ a predictive modeling approach to predict GCA based on fMRI task activation patterns during the *N*‐back working memory task as well as six other tasks in the Human Connectome Project dataset (*n* = 967), encompassing 15 task contrasts in total. We found tasks are a highly effective basis for prediction of GCA: The *2*‐*back* versus *0*‐*back* contrast achieved a 0.50 correlation with GCA scores in 10‐fold cross‐validation, and 13 out of 15 task contrasts afforded statistically significant prediction of GCA. Additionally, we found that task contrasts that produce greater frontoparietal activation and default mode network deactivation—a brain activation pattern associated with executive processing and higher cognitive demand—are more effective in the prediction of GCA. These results suggest a picture analogous to treadmill testing for cardiac function: Placing the brain in a more cognitively demanding task state significantly improves brain‐based prediction of GCA.

## Graphical abstract

We investigated prediction of general cognitive ability (GCA) based on fMRI task activation patterns with 15 task contrasts in the Human Connectome Project dataset. The *2*‐back versus *0*‐back contrast achieved a 0.50 correlation with GCA scores in ten10‐fold cross‐validation analysis. Additionally, we found that task contrasts that produce greater fronto‐parietal activation and default mode network deactivation—a brain activation pattern associated with executive processing and higher cognitive demand—are more effective in GCA prediction.

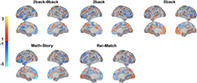

## 1 INTRODUCTION

In addition to particular abilities associated with individual cognitive tasks, there is substantial evidence for an overarching general ability involved in performance across a diverse range of tasks (Carroll, 2003; Horn & Noll, 1997; Mackintosh & Mackintosh, 2011; Neisser et al., 1996; Spearman, 1904). Test batteries composed of diverse tasks can yield accurate estimates of this general ability, which we here refer to as general cognitive ability (GCA) (Carroll, 1993; McGrew, 2009). GCA is a fundamental dimension of individual differences and is a key contributor to a number of important academic, occupational, health, and well‐being‐related outcomes (Batty, Mortensen, & Osler, 2005; Gale, Batty, Tynelius, Deary, & Rasmussen, 2010; Gottfredson, 1997; Ree, Earles, & Teachout, 1994; Strenze, 2007; Whitley et al., 2010). There is thus substantial interest in understanding the neural basis of GCA as well as the nature of inter‐individual neural differences.

Functional imaging studies of brain activation patterns during cognitive tasks have yielded important insights into the neural basis of GCA (Deary, Penke, & Johnson, 2010; Duncan et al., 2000; Gray, Chabris, & Braver, 2003; Schultz & Cole, 2016). In one key line of investigation, researchers identified a multiple demand network that activates across an array of cognitive tasks (Duncan, 2010; Duncan & Owen, 2000; Fedorenko, Duncan, & Kanwisher, 2013; Shashidhara, Mitchell, Erez, & Duncan, 2019). This network is hypothesized to support domain‐general functions such as working memory (Baddeley, 2012; D'Esposito, Postle, & Rypma, 2000) and cognitive control (Cole & Schneider, 2007; Miller & Cohen, 2001; Niendam et al., 2012) that contribute to performance across tasks irrespective of their specific content. Subsequent work found activation in key regions of this network, including dorsal lateral prefrontal cortex and superior parietal cortex, are correlated with measures of GCA or closely related constructs (DeYoung, Shamosh, Green, Braver, & Gray, 2009; Duncan et al., 2000; Gray et al., 2003; Lee et al., 2006).

A notable feature of many of these previous task‐based studies is that they are mainly concerned with localization and correlation: they mainly seek to identify specific brain regions whose activation correlates with GCA. Recently, however, another important goal has emerged in cognitive neuroscience: prediction (Rosenberg, Casey, & Holmes, 2018; Varoquaux & Poldrack, 2019; Yarkoni & Westfall, 2017). Unlike mass univariate approaches that are especially good for localization, predictive modeling approaches use multivariate methods that identify distributed patterns across the brain (“neurosignatures”). These distributed neurosignatures are often substantially more strongly related to phenotypes of interest than individual features because the neurosignatures aggregate information from across the entire brain (Woo, Chang, Lindquist, & Wager, 2017). However, because multivariate methods for constructing these distributed neurosignatures are highly parametrized, they are prone to overfitting. Predictive models are thus typically assessed by how well they predict unseen data, usually through the use of cross‐validation (Poldrack, Huckins, & Varoquaux, 2019; Scheinost et al., 2019).

Predictive modeling has been employed with a number of imaging modalities, including structural maps (Cox, Ritchie, Fawns‐Ritchie, Tucker‐Drob, & Deary, 2019) and resting‐state connectomes (Cui et al., 2020; Dubois, Galdi, Paul, & Adolphs, 2018; Finn et al., 2015; Sripada et al., 2019), to predict GCA or closely related constructs. A notable feature of these studies is that they mainly examined relatively stable, enduring features of the brain—features that are largely independent of the person's current cognitive state, and in particular their actual exercise of the cognitive abilities that are relevant to GCA. An alternative approach for building predictive models of GCA, which appears to be relatively less utilized (cf. Greene, Gao, Scheinost, & Constable, 2018; Stern, Gazes, Razlighi, Steffener, & Habeck, 2018), employs a rationale similar to that for cardiac treadmill testing. This approach attempts to first place the brain in an activated state that engages the cognitive abilities associated with GCA. By activating the brain in this way, individual differences in the neural basis of GCA may be rendered more “visible” for a predictive model to detect (see Finn et al. 2017 for a suggestion along these lines).

In the current study, we adopted this second approach. Utilizing the Human Connectome Project's (HCP) 1200 release, we began by constructing a highly reliable measure of GCA from 10 measures from the NIH Toolbox and Penn Neurocognitive Battery (Dubois et al., 2018). We then used a predictive modeling framework to examine the prediction of GCA from contrast maps derived from the *N*‐back working memory task as well as six other fMRI tasks (15 task contrasts in total). We demonstrate two things. First, task‐based brain activation patterns allow highly reliable prediction of GCA, with performance appreciably higher than that typically reported in other neuroimaging modalities. Second, tasks that produce greater frontoparietal activation and default mode network (DMN) deactivation, which is associated with higher cognitive demand, are more effective at GCA prediction.

## 2 METHODS

### 2.1 Subjects and data acquisition

All subjects and data were from the HCP‐1200 release (Van Essen et al., 2013; WU‐Minn HCP, 2017) and all research was performed in accordance with relevant guidelines and regulations. Subjects provided informed consent, and recruitment procedures and informed consent forms, including consent to share de‐identified data, were approved by the Washington University institutional review board. Subjects completed two runs each of seven scanner tasks across two fMRI sessions, using a 32‐channel head coil on a 3T Siemens Skyra scanner (TR = 720 ms, TE = 33.1 ms, 72 slices, 2 mm isotropic voxels, multiband acceleration factor = 8) with right‐to‐left and left‐to‐right phase encoding directions. Comprehensive details are available elsewhere on HCP's overall neuroimaging approach (Glasser et al., 2013; Van Essen et al., 2013) and HCP's task fMRI dataset (Barch et al., 2013).

For the construction of a GCA factor, all subjects with available data were included. This analysis included 1,192 subjects. For the brain imaging analysis, subjects were eligible to be included if they had available task data in MSMAll format [information about both folding as well as function are used for cross‐subject alignment (Glasser et al., 2016)] for both runs of all seven tasks, had full behavioral data, and no more than 25% of their volumes in each run exceeded a framewise displacement threshold of 0.5 mm. These exclusions resulted in a sample of 967 subjects.

### 2.2 Data preparation

Data were preprocessed through the HCP minimally preprocessed pipeline, which is presented in detail by Glasser et al. 2016. Briefly, the pipeline includes gradient unwarping, motion correction, fieldmap distortion correction, brain‐boundary based linear registration of functional to structural images, nonlinear registration to MNI152 space, and grand‐mean intensity normalization. Data then entered a surfaced‐based preprocessing stream, followed by grayordinate‐based processing, which involves data from the cortical ribbon being projected to surface space and combined with subcortical volumetric data.

### 2.3 fMRI tasks

We used contrasts from seven HCP tasks, described in brief in Table 1 [detailed descriptions are available elsewhere (Barch et al., 2013; WU‐Minn HCP, 2017)].

**TABLE 1:** Seven human connectome project fMRI tasks

N‐back task	Participants respond when the picture shown on the screen is the same as the one two trials back (*=2‐back* condition) or the same as one shown at the start of the block (*=0‐back* condition)
Incentive processing	Participants guess whether the number on a mystery card will be more or less than 5 and win or lose money (*reward* condition = mostly wins; *loss* condition = mostly losses)
Motor	Participants move fingers, toes, and tongue
Language task	Participants answer questions about Aesop's fables (=*story* condition) or math problems (=*math* condition)
Social cognition task	Participants watch video clips of objects interacting in an agentive way (=*theory of mind* condition) or random way (=*random* condition)
Relational task	Participants identify the dimension along which a cue pair of objects differs and determine if a target pair differs along the same dimension (=*relational* condition). Or they determine if a cue object matches a member of a target pair along a given dimension (=*match* condition)
Emotion task	Participants decide whether one of two presented faces match one at the top of the screen (=*face* condition) or else they perform the same task with shapes (=*shape* condition)

At the single subject‐level, fixed‐effects analyses were conducted using FSL's FEAT to estimate the average effects across runs within‐participants, using 2 mm surface smoothed data. Some tasks permitted multiple contrasts beyond the standard experimental versus control condition (e.g., *N*‐back allows additional contrasts based on all four stimulus types). To reduce the complexity of the analysis and avoid loss of power from a smaller number of trials, we focused on the standard contrasts associated with these tasks. The Language Task and Emotion Task lacked fixation blocks. Thus, we included the main condition contrasts (e.g., math‐story and faces‐shapes), but we did not include each of these conditions versus baseline. A full list of filenames of the contrast maps used can be found in Table S1.

### 2.4 Constructing a GCA factor

We conducted an exploratory factor analysis utilizing the strategy and associated code made available by Dubois and colleagues (https://github.com/adolphslab/HCP_MRI-behavior), who recently investigated the prediction of GCA from resting‐state fMRI in the HCP dataset (Dubois et al., 2018). Unadjusted scores from 10 cognitive tasks for 1,181 HCP subjects were included in the analysis (subjects with missing data or MMSE <26 were excluded), including seven tasks from the NIH Toolbox (Dimensional Change Cart Sort, Flanker Task, List Sort Test, Picture Sequence Test, Picture Vocabulary Test, Pattern Completion Test, Oral Reading Recognition Test), and three tasks from the Penn Neurocognitive Battery (Penn Progressive Matrices, Penn Word Memory Test, Variable Short Penn Line Orientation Test), with additional details supplied in Dubois et al. (2018).

We applied Dubois and colleagues' code to this data, which uses the omega function in the psych (v 1.8.4) package (Revelle, 2016) in R (v3.4.4). In particular, the code performs maximum likelihood‐estimated exploratory factor analysis (specifying a bifactor model), oblimin factor rotation, followed by a Schmid–Leiman transformation (Schmid & Leiman, 1957) to find general factor loadings.

To assess reliability, in a separate analysis, we re‐ran the factor analysis excluding 46 subjects that had test/retest sessions available. We then estimated factor scores for both sessions for these subjects and calculated test/retest reliability via intraclass correlation [we used ICC (2,1) in the Shrout and Fleiss scheme (Shrout & Fleiss, 1979)].

We performed the preceding factor analysis on the entire dataset to characterize the factor structure (see Section 3.1). But importantly, we in addition repeated the factor analysis multiple times, each time within a fold of a 10‐fold cross‐validation analysis (see Section 2.6). This was to ensure the complete separation of train and test datasets during cross‐validation.

### 2.5 Brain basis set modeling

**FIGURE 1:**
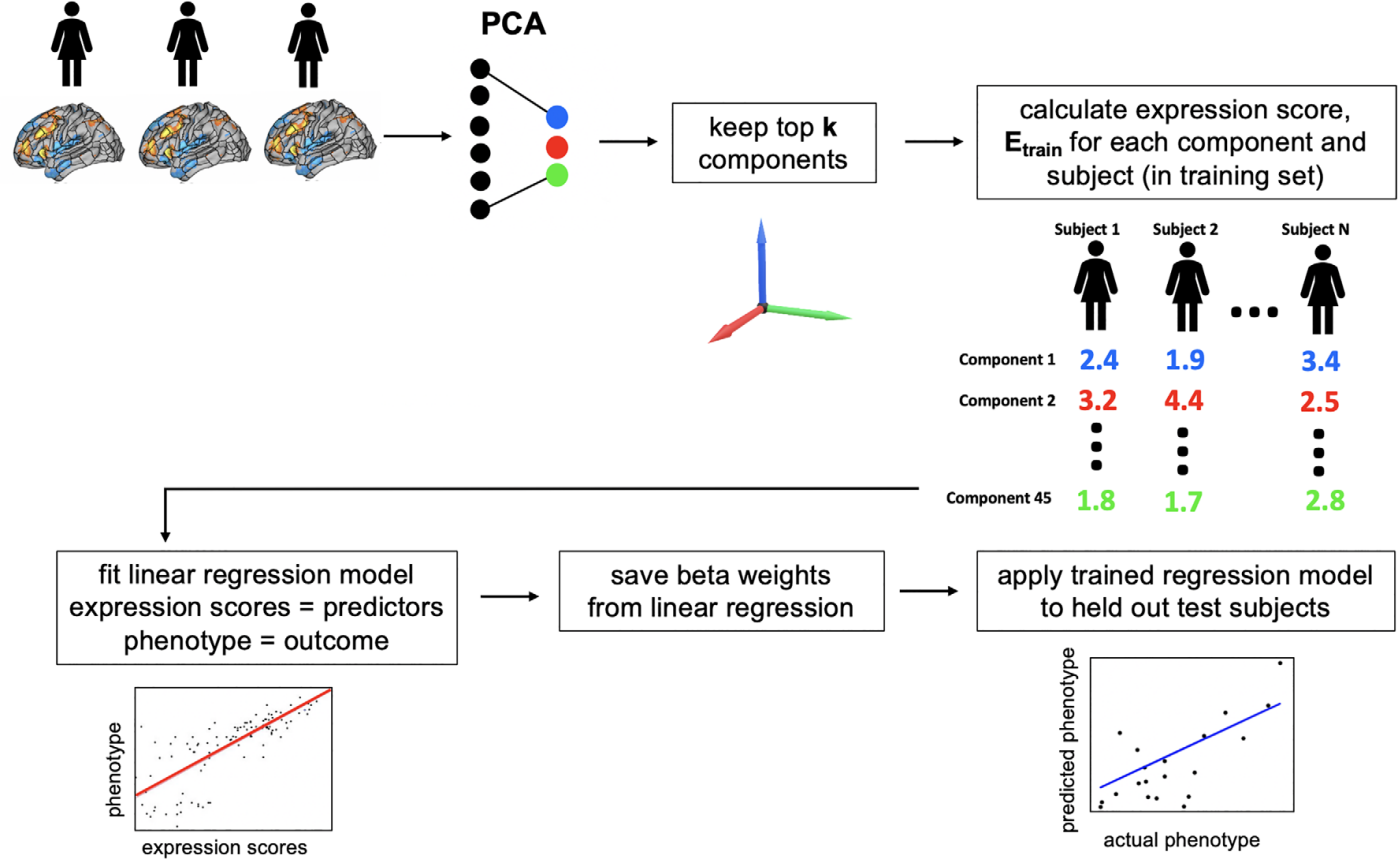
Main steps of brain basis set (BBS) modeling. BBS is a multivariate predictive modeling method. It utilizes dimensionality reduction with principal components analysis (PCA) to construct a basis set for predicting phenotypes of interest

Our aim was to predict each subject's GCA scores from each of the 15 task contrasts. To accomplish this, we used Brain Basis Set (BBS) modeling, previously described in detail (Sripada, Angstadt, Rutherford, Kessler, et al., 2019; Sripada, Rutherford, Angstadt, Thompson, et al., 2019) and presented here in brief (Figure 1). Note that BBS was applied separately to each of the 15 task contrasts, and thus the steps that follow are performed separately for each contrast.

BBS assumes a train/test split of the dataset (see Section 2.6 below). In the train dataset, each subject's task contrast was vectorized and then concatenated yielding an *n* subjects × *m* voxels matrix. This matrix was then submitted to principal components analysis using the pca function in MATLAB (2015b), yielding *n*‐1 components ordered by descending eigenvalues, of which we retained the top 75 components.

We selected 75 as the number of components to retain based on prior analysis in which we estimated the number of intrinsic dimensions associated with each task contrast. This was accomplished by submitting each of the task contrast matrices to the dimensionality estimation procedure of Levina & Bickel (2004). This is a maximum likelihood estimation method based on the distance between close neighbors, which we previously successfully applied to HCP resting‐state data (Sripada, Angstadt, Rutherford, Kessler, et al., 2019). Dimensionality estimation found a mean of 72 dimensions across the 15 task contrasts. Because prior studies by our group (Sripada, Angstadt, Rutherford, Kessler, et al., 2019) showed small differences in the number of components make little difference in classifier performance, and to increase comparability with recent studies that used 75 components (Sripada, Angstadt, Rutherford, Kessler, et al., 2019; Sripada, Rutherford, Angstadt, Thompson, et al., 2019), we chose to use 75 components for each task.

Next, in the training dataset, we calculate the expression scores for each of the components for each subject by projecting their data onto the 75 principal components. We then fit a linear regression model with these expression scores as predictors and the phenotype of interest (i.e., GCA) as the outcome, saving **B**, the 75 × 1 vector of fitted coefficients, for later use. In a test dataset, we again calculate the expression scores for each of the 75 components for each subject. Our predicted phenotype for each test subject is the dot product of **B** learned from the training dataset with the vector of component expression scores for that subject.

### 2.6 10‐fold cross‐validation

To assess the performance of BBS‐based prediction models, we used 10‐fold cross‐validation. Because there is family structure in the HCP dataset, we ensured that family members always appeared within a single partition (and thus in no cases was the BBS classifier trained on a member of a family and tested on another member of that family).

To ensure complete separation of the train and test datasets, in each fold of the cross‐validation, we did the following in the train dataset: First, a PCA was performed on the task contrast yielding a 75‐component basis set. Second, the exploratory factor analysis described in Section 2.2 was performed yielding GCA scores for each train subject. In addition, the betas representing factor loadings for each behavioral task were applied to the test dataset, yielding GCA scores for the test subjects.

### 2.7 Accounting for covariates in a cross‐validation framework

In each fold of cross‐validation, BBS models were trained in the train partition with the following covariates (similar to Dubois et al., 2018): age, age squared, handedness, gender, brain size, multiband reconstruction algorithm version number (HCP variables: Age_In_Yrs, Handedness, Gender, FS_BrainSeg_Vol, fMRI_3T_ReconVrs), and mean framewise displacement (mean FD; task‐specific values were used) and mean FD squared. Thus, our generative model for the data had the following form:

(1) $$y_{\text{train}}=X_{\text{train}}\beta +Z_{\text{train}}\gamma +\varepsilon$$

where *y*_train_ is the train set response variable, *X*_train_ is the train set brain features design matrix, *β* is the train set brain features regression coefficients, *Z*_train_ is the train set covariate design matrix, *γ* is the train set covariate regression coefficients, and *ε* is Gaussian mean zero error.

When this model is estimated, we are particularly interested in the relationship between the following two terms:

(2) $$y_{\text{train}}-Z_{\text{train}}\hat{\gamma }$$

(3) $$X_{\text{train}}\hat{\beta }$$

where $\hat{\gamma }$ is the estimated train set covariate regression coefficients and $\hat{\beta }$ is the estimated train set brain features regression coefficients. Term (2) represents the response variable adjusted for the estimated effects of the nuisance covariates, while term (3) represents the prediction of this covariate‐adjusted response variable based on brain features. To be clear, $X_{\text{train}}\hat{\beta }$ is a prediction of the *covariate‐adjusted* response because $\hat{\beta }$ is learned in a model with covariates.

To assess this same relationship in the test dataset, we compute quantities analogous to (2) and (3) in the test dataset. But to maintain the strict separation between train and test datasets needed in cross‐validation, we compute these quantitates using the coefficients learned in the train dataset. Thus, we examine the relationship between

(4) $$y_{\text{test}}-Z_{\text{test}}\hat{\gamma }$$

(5) $$X_{\text{test}}\hat{\beta }$$

where *Z*_test_ is the test set design matrix, $\hat{\gamma }$ is the covariate regression coefficients learned from the train dataset, *X*_test_ is the test set brain features design matrix, and $\hat{\beta }$ is the brain features regression coefficients learned from the train dataset.

### 2.8 Evaluation of cross‐validation performance

Overall performance across the 10‐fold cross‐validation was assessed in three ways. Our primary measure is based on the correlation between the observed covariate‐adjusted outcome variable and predicted outcome variable:

$$\text{correlation}\left({\tilde{y}}_{\text{test}},{\hat{y}}_{\text{test}}\right)$$

where ${\tilde{y}}_{\text{test}}\;$ is term (4) above, that is, the test set response variable adjusted for covariates based on coefficients learned in the train dataset, and ${\hat{y}}_{\text{test}}$ is term (5) above, that is, the predicted covariate‐adjusted response variable for the test set. Correlations were computed for each fold. To obtain the average correlation across folds, the per‐fold correlations were Fisher *r* to *z* transformed, the transformed correlations were averaged across all folds, and then this average was *z* to *r* transformed. Confidence intervals were estimated as 95% at intervals based on the mean and *SD* over cross‐validation folds.

In addition, we report a cross‐validated coefficient of determination $R_{\mathrm{cv}}^{2}$ and mean square error (MSE), which are calculated as follows:

$$R_{\mathrm{cv}}^{2}=1-\frac{\sum_{i=1}^{n}{\left({\tilde{y}}_{i}-{\hat{y}}_{i}\right)}^{2}}{\sum_{i=1}^{n}{\left({\tilde{y}}_{i}-\bar{y}\right)}^{2}}$$

$$\mathrm{MSE}=\frac{1}{n-1}\sum_{i=1}^{n}{\left({\tilde{y}}_{i}-{\hat{y}}_{i}\right)}^{2}$$

where ${\tilde{y}}_{i}$ is the covariate‐adjusted response variable for the test set for subject *i*, ${\hat{y}}_{i}$ is the predicted covariate‐adjusted response variable for the test set for subject *i*, $\bar{y}$ the mean value of the response variable for the train set, and *n* is the number of test set subjects. We calculate these values for each fold and then average across folds.

### 2.9 Permutation tests

To assess the statistical significance of BBS models, we used nonparametric permutation methods. The distribution under chance of correlations between BBS‐based predictions of neurocognitive scores and observed neurocognitive scores was generated by randomly permuting the subjects' neurocognitive scores 10,000 times. At each iteration, we performed the 10‐fold cross‐validation procedure described above, which includes refitting BBS models at each fold of the cross‐validation. We then recalculated the average correlation across folds between predicted versus actual neurocognitive scores. The average correlation across folds that was actually observed was located in this null distribution in terms of rank, and statistical significance was set as this rank value divided by 10,000.

Since the BBS models fit at each iteration of the permutation test included covariates, the procedure of Freedman and Lane was followed (Freedman & Lane, 1983). In brief, a BBS model was first estimated with nuisance covariates alone, residuals were formed and were permuted. The covariate effect of interest was then included in the subsequent model, creating an approximate realization of data under the null hypothesis, and the statistical test of interest was calculated on this data (see FSL Randomise http://fsl.fmrib.ox.ac.uk/fsl/fslwiki/Randomise/Theory for a neuroimaging implementation).

### 2.10 Consensus predictive maps for visualization

We used BBS with 75 whole‐brain components to make predictions about GCA. To help convey overall patterns across the entire BBS predictive model, we constructed “consensus” predictive maps. We first multiplied each component map with its associated beta from the fitted BBS model. Next, we summed across all 75 components yielding a single map, and *z* scored the entries.

### 2.11 Analysis of resting‐state connectomes

To help contextualize results from predictive modeling applied to task contrast data, we applied this same predictive modeling stream to resting‐state connectomes. Data used were from the HCP‐1200 release (Van Essen et al., 2013; WU‐Minn HCP, 2017). Four runs of resting‐state fMRI data (14.4 min each; two runs per day over 2 days) were acquired using the same acquisition sequence described above in Section 2.1. Processed volumetric data from the HCP minimal preprocessing pipeline including ICA‐FIX denoising were used. Full details of these steps can be found in Glasser et al. (2013) and Salimi‐Khorshidi et al. (2014).

Data then went through a number of resting‐state processing steps, including a motion artifact removal steps comparable to the type B (i.e., recommended) stream of Siegel et al. (2017). These steps include linear detrending, CompCor to extract and regress out the top five principal components of white matter and CSF (Behzadi, Restom, Liau, & Liu, 2007), bandpass filtering from 0.1 to 0.01 Hz, and motion scrubbing of frames that exceed a framewise displacement of 0.5 mm. We next calculated spatially averaged time series for each of 264 4.24 mm radius regions of interest (ROIs) from the parcellation of Power et al. (2011). We then calculated Pearson's correlation coefficients between each ROI. These were then transformed using Fisher's *r* to *z* transformation.

Subjects consisted of those subjects included in the main task contrast analysis who had four complete resting‐state fMRI runs (14 m 24 s each). In addition, subjects with more than 10% of resting‐state frames censored were excluded. This resulted in 903 subjects who entered a BBS predictive modeling analysis for prediction of GCA scores using the same BBS approach that is described above.

## 3 RESULTS

### 3.1 Constructing a GCA factor from 10 HCP behavioral tasks

**FIGURE 2:**
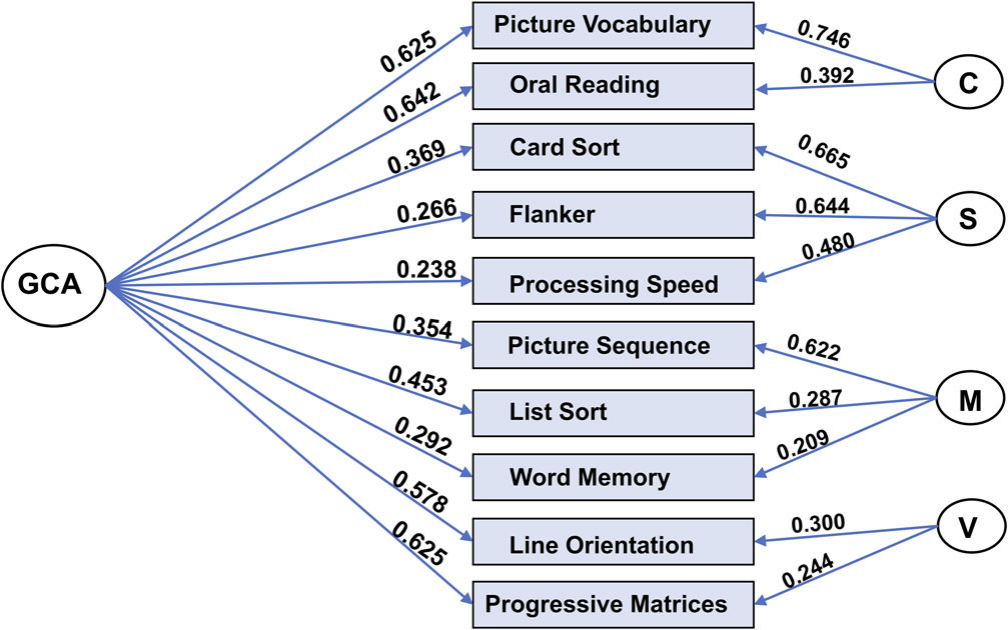
Bifactor model of general cognitive ability. We performed the bifactor exploratory factor analysis on 10 behavioral tasks in the human connectome project (HCP) dataset. The resulting model consisted of a general factor (“GCA”) and four group factors and exhibited an excellent fit with the data. C, crystallized cognitive ability; S, processing speed; M, memory; V, visuospatial ability

We began by fitting a bifactor model to the behavioral data for the entire dataset. Similar to the findings of Dubois et al. (2018) who examined a largely overlapping set of subjects, this model fit the data very well (CFI = 0.989; RMSEA = 0.036; SRMR = 0.0200; BIC = 0.782). The solution, which included a general factor and four group factors, is depicted in Figure 2. Similar to Dubois and colleagues, we interpret the four group factors as: (a) crystallized ability, (b) processing speed, (c) memory, and (d) visuospatial ability.

The general factor, which we refer to throughout as the GCA factor and which is the focus of this report, accounts for 58.6% of the variance [coefficient omega hierarchical ω (Zinbarg, Revelle, Yovel, & Li, 2005)], while group factors account for 18.0% of the variance. Based on the 46 subjects in the retest dataset for HCP, test–retest reliability for GCA was found to be 0.78, which is conventionally classified as very good [we used ICC (2,1) in the Shrout and Fleiss scheme (Shrout & Fleiss, 1979)].

### 3.2 Contrasts associated with the *N*‐Back task are highly effective at predicting GCA

Because working memory has been strongly and consistently linked with GCA (Conway, Kane, & Engle, 2003; Duncan, Schramm, Thompson, & Dumontheil, 2012; Engle et al., 2001; Engle & Kane, 2004,Kyllonen and Christal, 1990) we first investigated the prediction of GCA based on the *N*‐back working memory task. We used BBS modeling with 75 components and a 10‐fold cross‐validation procedure. The average correlation across folds between predicted GCA and actual GCA was 0.50, which was highly statistically significant (permutation‐based *p* < .0001, observed correlation was higher than all 10,000 in the permutation distribution).

**FIGURE 3:**
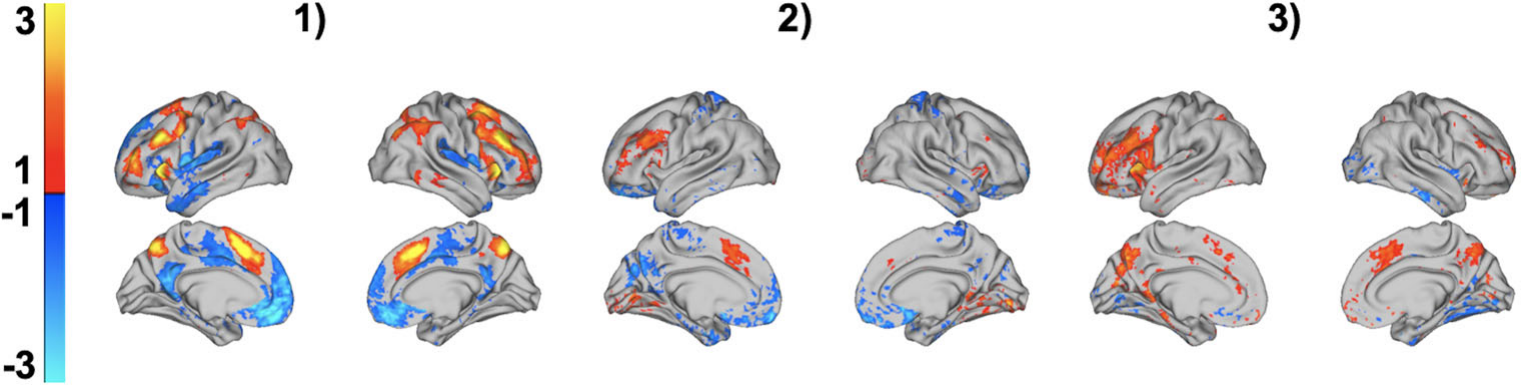
Visualization of the three components from the *2‐Back* versus *0‐Back* task contrast most predictive of general cognitive ability (GCA). We found the 2‐back versus 0‐back contrast was highly effective for GCA prediction, achieving a 0.50 correlation with GCA scores in 10‐fold cross‐validation. From a 75‐component brain basis set model trained to predict GCA scores, the three most statistically significant components are shown above

Figure 3 shows the top three components based on statistical significance displayed so that greater expression of these components predicts higher GCA. These components include large activations in the supplementary motor area (SMA), precuneus, and dlPFC, as well as deactivations in anterior DMN. To convey “average” patterns across all 75 components, we constructed consensus predictive maps (see Section 2) and they are displayed in Figure 3. These show additional patterns predictive of GCA, including deactivation of the posterior cingulate cortex and frontopolar cortex.

**FIGURE 4:**
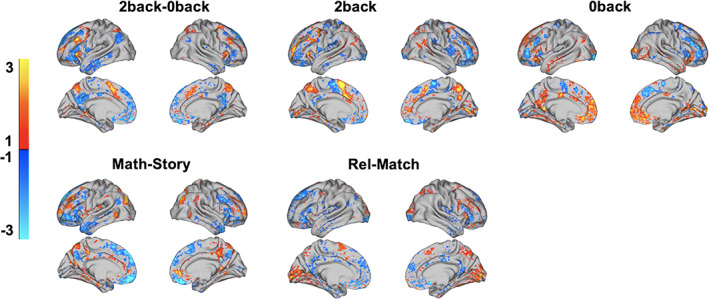
Consensus predictive maps for five task contrasts highly predictive of general cognitive ability (GCA). We found 13 out of 15 task contrast maps yielded highly statistically significant predictions of GCA in 10‐fold cross‐validation analysis. For the five most predictive task contrasts, we constructed consensus predictive maps that display brain activation patterns that were most predictive of GCA. Rel, relational

We next trained additional BBS models on the *2*‐*back* versus *baseline* and *0*‐*back* versus *baseline* contrasts. The correlation across folds of the 10‐fold cross‐validation procedure between predicted GCA and actual GCA was 0.48 and 0.35, respectively. The consensus predictive maps, shown in Figure 4, revealed an interesting change in directionality across these contrasts. For example, pre‐SMA strongly predicts higher GCA in the *2*‐*back* contrast versus *baseline* but the reverse is true in the *0*‐*back* versus *baseline* contrast. Additionally, less activation (i.e., deactivation) of the anterior DMN predicts higher GCA in the *2*‐*back* versus *baseline* contrast, but the reverse is true in the *0*‐*back* versus *baseline* contrast.

### 3.3 Looking across all 15 task contrasts, tasks involving more executive processing and higher cognitive demand are more effective in predicting GCA

We next examined the remaining 12 contrasts from the other six HCP tasks. As with the *N*‐back task, we constructed BBS models predicting GCA scores from each contrast, and assessed the performance of these models in 10‐fold cross‐validation analysis.

**FIGURE 5:**
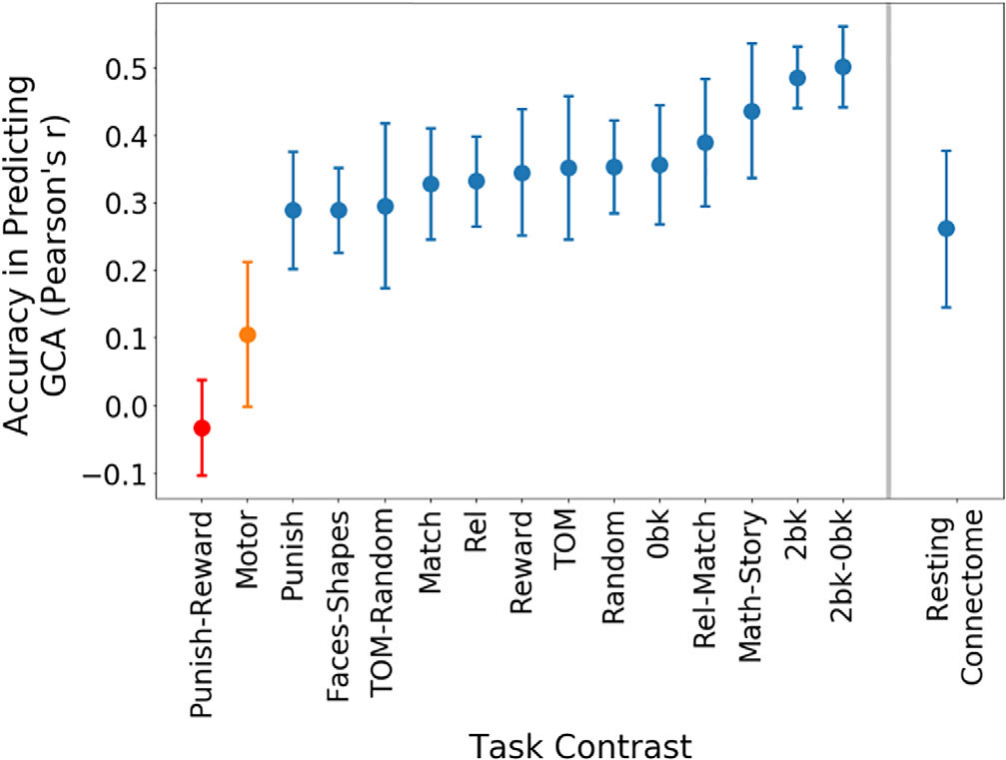
Prediction of general cognitive ability (GCA) across 15 task contrasts. We used the brain basis set (BBS) predictive modeling approach to predict GCA from each of the 15 human connectome project (HCP) task contrasts. The *y*‐axes in the figure refer to the accuracy of these BBS models in predicting GCA, as measured by the correlation between observed and predicted GCA scores in 10‐fold cross‐validation. For comparison, we additionally plot accuracy of GCA prediction using BBS methods applied to another modality: resting‐state connectomes. Error bars represent the 95% confidence interval; blue = permutation‐based *p*‐value <.0001, observed correlation was higher than all 10,000 in the permutation distribution; orange = permutation‐based *p*‐value <0.05; red = permutation‐based *p*‐value is not significant. TOM, theory of Mind; Rel, Relational

The results are shown in Figure 5 and Table 2. Using permutation‐based statistical testing with 10,000 permutations, we found that 13 out of the 15 task contrasts produced statistically significant predictions of GCA (shown in blue and orange in Figure 5). The *2‐back* versus, *0‐back* contrast was the most effective single task contrast for GCA prediction, achieving a 0.50 correlation with GCA scores in 10‐fold cross‐validation. Other tasks involving executive processing were top performers, including the *relational* versus *match* contrast from the relational processing task and the *math* versus *story* contrast from the language‐processing task. Resting‐state connectomes yielded prediction accuracy of *r* = .26. In comparison, 13 out of 15 task contrasts performed better.

**TABLE 2:** Prediction of general cognitive ability (GCA) across 15 task contrasts

Task	$R_{\mathrm{cv}}^{2}$	Mean squared error (MSE)
2back‐0back	0.280	0.576
2back	0.265	0.604
Math‐Story	0.227	0.623
Rel‐Match	0.195	0.639
Random	0.172	0.658
0back	0.172	0.659
TOM	0.172	0.654
Reward	0.165	0.662
Rel	0.156	0.664
Match	0.155	0.669
Punish	0.132	0.686
TOM‐Random	0.130	0.692
Faces‐Shapes	0.127	0.691
Motor	0.049	0.751
Punish‐Reward	0.033	0.763
Resting connectome	0.078	0.755

*Note*: We used the brain basis set (BBS) predictive modeling approach to predict GCA from each of 15 HCP task contrasts. For comparison, we additionally include results from predicting GCA with resting‐state connectomes. The table shows model performance assessed with cross‐validated *r* squared ($R_{\mathrm{cv}}^{2}$) and mean square error (MSE).

Abbreviations: TOM, theory of mind; Rel, relational.

### 3.4 Mean activation levels of FPN and DMN predict which task contrasts are effective for GCA prediction

**FIGURE 6:**
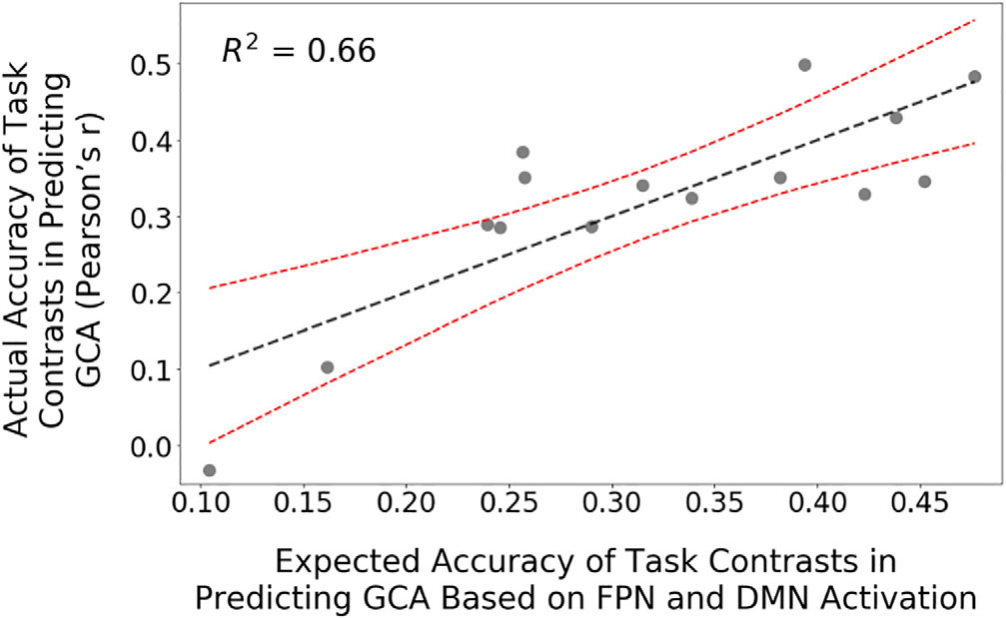
Frontoparietal network (FPN) and default mode network (DMN) activation patterns and effectiveness of task contrasts in predicting general cognitive ability (GCA). We hypothesized that placing the brain in an activated, cognitively demanding state improves the prediction of GCA. We thus calculated FPN and DMN activation levels, which are thought to index cognitive demandingness, for each of the 15 task contrasts. We in addition calculated each of the 15 task contrast's accuracy in predicting GCA, as measured by the correlation between observed and predicted GCA scores in 10‐fold cross‐validation. In multiple regression analysis, we found that FPN/DMN activation levels for the 15 contrasts (*x*‐axis) were indeed strongly related to the contrasts' accuracy in predicting GCA (*y*‐axis). That is, contrasts that activated FPN/deactivated DMN more afforded higher accuracy in predicting GCA. Red dashed lines represent the 95% confidence interval

A number of studies have observed that tasks that are cognitively demanding produce activation in regions of frontoparietal network (FPN) (Cabeza & Nyberg, 2000; Cole & Schneider, 2007; Duncan & Owen, 2000; Niendam et al., 2012) and deactivation of regions of DMN (Anticevic et al., 2012; Anticevic, Repovs, Shulman, & Barch, 2010; Esposito et al., 2006; McKiernan, Kaufman, Kucera‐Thompson, & Binder, 2003). Building on these observations, we hypothesized that more cognitively demanding task contrasts (operationalized in terms of activation levels of FPN and DMN) should be more effective in predicting GCA. We extracted mean activation across the seven networks in Yeo and colleagues' parcellation (Yeo et al., 2011) and examined correlations with the accuracy of GCA prediction across the 15 task contrasts (prediction accuracy is measured with the cross‐validated correlation between observed and predicted GCA scores). We found that FPN activation was indeed strongly and statistically significantly related to the accuracy of GCA prediction (*r* = .68, *p* = .006). DMN activation was also (inversely) related to the accuracy of GCA prediction (*r* = −.20), but the correlation did not reach statistical significance. We also created a regression model in which both FPN and DMN activation jointly predict the accuracy of GCA prediction. The correlation across task contrasts between fitted predictions from the regression model and actual accuracy in predicting GCA was *r* = .82 (*p* = .001; Figure 6). None of the other five Yeo networks was statistically significantly related to GCA prediction.

### 3.5 Across the 15 task contrasts, activation signatures of GCA are spatially distributed and task‐specific

We next compared the consensus predictive maps associated with the 15 contrasts (five maps are shown in Figure 4, and the remaining maps are shown in Figure S1). Signatures for predicting GCA associated with each task were highly distributed, with notable variation in these signatures across tasks. Prominently represented regions include superior parietal cortex (*reward* vs. *baseline*, *punishment* vs. *baseline*), dlPFC (*math* vs. *story*), anterior insula (*relational* vs. *match*), frontopolar cortex (*math* vs. *story*), pre‐SMA (*relational* vs. *match*), and visual cortex (*relational* vs. *match*, *reward* vs. *baseline*, *punishment* vs. *baseline*).

## 4 DISCUSSION

Task‐based imaging provides a promising route for constructing brain‐based predictive models of GCA because tasks can potentially selectively activate brain regions responsible for effective cognitive performance. Thus, we systematically assessed neuroimaging‐based prediction of GCA from 15 fMRI task conditions in the HCP dataset. Our first main finding is that whole‐brain task activation patterns are a highly effective basis for prediction of GCA, with a model trained on activation during the *N*‐back working memory task (*2‐back* vs. *0‐back* contrast) achieving a 0.50 correlation with GCA scores in 10‐fold cross‐validation. Our second main finding is that more cognitively demanding tasks that more vigorously activate FPN and deactivate DMN are particularly effective for GCA prediction. These results highlight the utility of placing the brain in a cognitively demanding, activated task state for improved brain‐based prediction of GCA.

### 4.1 Role of executive regions in prediction of GCA

The importance of FPN, as well as related executive regions (e.g., dorsal anterior cingulate), for GCA has been highlighted in previous work, especially in Jung and Haier's influential frontoparietal integration theory (Jung & Haier, 2007). In a similar vein, Duncan, Owen, Fedorenko, and colleagues have proposed that “multiple demand” cortex—regions of the brain that activate across a broad range of cognitively demanding tasks (Duncan, 2010; Duncan & Owen, 2000; Fedorenko et al., 2013; Shashidhara et al., 2019)—are a primary substrate of GCA (Duncan et al., 2000). The present study extends these findings using a multivariate predictive modeling framework that identifies distributed neurosignatures across the brain that are predictive of GCA. We showed that executive regions are important in these distributed neurosignatures in three complementary ways.

First, in looking across the set of 15 contrasts derived from seven HCP tasks, we found that tasks that tap executive processes were more predictive of GCA (e.g., *N*‐back *2‐back* vs. *0‐back* contrast, relational reasoning *relational* vs. *match* contrast, and *math* vs. *story* contrast). Second, we found that FPN activation and DMN deactivation, highly associated with the cognitive demandingness of task conditions (Anticevic et al., 2010; Anticevic et al., 2012; Cabeza & Nyberg, 2000; Cole & Schneider, 2007; Duncan & Owen, 2000; Esposito et al., 2006; McKiernan et al., 2003; Niendam et al., 2012), predicts which task contrasts will be effective for GCA prediction. Third, within highly predictive contrasts, such as the *2‐back* versus *0‐back* contrast and *math* versus *story* contrast, activation patterns in executive regions were prominent among regions predictive of GCA.

Overall, the N‐back *2‐back* versus *0‐back* contrast performed best in GCA prediction. This is consistent with the finding that working memory is highly related to GCA (Duncan et al., 2012; Engle et al., 2001; Engle & Kane, 2004). However, the differences in performance between the three main executive task contrasts—that is, *2‐back* versus *0‐back*, *math* versus *story*, and *relational* versus *match*—were modest. Future studies with larger sample sizes should investigate whether all executive tasks are similarly effective with respect to GCA prediction, which would align well with the multiple demand network hypothesis. Or alternatively, there are subtle differences across executive tasks in affording GCA prediction.

Interestingly, for certain regions, the directionality of prediction of GCA exhibited some variability across task contrasts in a way suggestive of moderation by task difficulty (e.g., see pre‐SMA in *0‐back* compared to *2‐back* and in *match* compared to *relational*; we discuss moderation by the cognitive load in these tasks further in Sripada, Angstadt, Rutherford, & Taxali 2019). These observations are consistent with a neural efficiency model of GCA proposed by Neubauer & Fink (2009). They propose that higher GCA is associated with greater processing efficiency in elementary cognitive tasks (leading to less activation in higher GCA individuals) but greater processing capacity in demanding cognitive tasks (leading to greater activation in higher GCA individuals), thus potentially explaining the flipped directions of activation observed across the easy and hard conditions of the N‐back and other tasks.

While activation patterns in executive regions clearly play an important role in explaining the success of our task‐based approach to GCA prediction, there is still clear evidence for discriminative information about GCA located outside executive regions. This is apparent in looking at the consensus predictive maps for each of the 15 task contrasts in Figure 4 as well as Figure S1. Non‐executive regions, such as the lateral temporal cortex and temporal pole, are found in several of these consensus maps, indicating they too are important for the prediction of GCA.

### 4.2 Comparison of task‐based prediction with other modalities

Previous studies have examined correlations between GCA and structural brain imaging features including cortical thickness (Colom et al., 2009; Shaw et al., 2006) and white matter structure (Turken et al., 2008), for reviews see Deary et al. (2010), Jung & Haier (2007), and Luders, Narr, Thompson, & Toga (2009). It is notable that the correlations reported with these modalities tend to be modest. For example, the correlations with brain volume, one of the most studied variables, are typically reported to be between 0.1 and 0.3 (McDaniel, 2005; Pietschnig, Penke, Wicherts, Zeiler, & Voracek, 2015). In terms of functional MRI, recent studies have examined resting‐state connectivity patterns (Dubois et al., 2018; Finn et al., 2015; Sripada, Angstadt, Rutherford, Kessler, et al., 2019; Sripada, Rutherford, Angstadt, Thompson, et al., 2019). In the present study, we found resting‐state connectomes, which entered the same BBS prediction pipeline as our task‐based contrast maps, achieved a correlation of 0.26 with GCA [broadly similar to the results from our recent study using BBS modeling to predict neurocognition from resting‐state connectomes in 2,013 youth (Sripada, Rutherford, Angstadt, Thompson, et al., 2019)]. These results, however, are appreciably smaller than the 0.50 correlation we found when applying BBS predictive modeling to the *2‐back* versus *0‐back* task contrast in the present study.

There are two interrelated reasons why task‐based fMRI might potentially offer a more reliable prediction of GCA than other imaging modalities. The first appeals to the “treadmill testing” idea already mentioned: actively engaging in cognitive tasks has the potential to unmask critical GCA‐relevant features of the brain that are otherwise invisible in other modalities such as structural or resting‐state brain imaging (Finn et al., 2017; Greene et al., 2018). A second potential advantage of task‐based methods is specificity. Tasks are constructed by their designers to target specific psychological processes, often with control conditions that subtract away contributions from auxiliary processes of no interest. This will tend to make classification more accurate as the feature set is culled of a sizable number of uninformative features.

### 4.3 Future directions

While we found strong predictivity of GCA from fMRI task contrasts, even the strongest performing task contrast explained only 28% of the variance (*R*_cv_) in GCA scores. Thus, the majority of variance in GCA scores remains to be explained, which raises the question of how we might improve performance in future studies. In considering this question, it is notable that we used the set of imaging tasks that were included in the HCP dataset. These imaging tasks, in turn, were selected based on diverse considerations (see Barch et al. 2013), but maximizing the prediction of GCA was not among them. Thus, it is plausible that one can do still better: It should be possible to intentionally design and optimize an imaging task battery to yield even more accurate task‐based prediction of GCA.

Given our observation that tasks that more vigorously activate FPN and deactivate DMN afford better prediction of GCA, a natural approach is to focus on highly demanding tasks that produce this activation profile. One natural candidate is an N‐back task with increased cognitive load [e.g., a 3‐back (Braver et al., 1997; Pochon et al., 2002) or 4‐back task] Other executive function tasks, such as tasks involving response inhibition, task switching, or higher‐order reasoning, are also plausible. Moreover, it is possible that task contrasts from an executive task battery, as opposed to a contrast from a single task, could afford still better GCA prediction.

In sum, this study firmly establishes the effectiveness of task‐based fMRI for prediction of GCA and demonstrates that tasks that are more cognitively demanding are associated with better prediction accuracy.

## CONFLICT OF INTEREST

The authors declare no potential conflict of interest.

## Supporting information


**Appendix** **S1.** Supporting Information.

## Data Availability

Human Connectome Project data are publicly available to the research community at the project website: http://www.humanconnectomeproject.org/. Consensus component maps for all predictive models associated with each of the 15 task contrasts have been shared on BALSA, the Human Connectome Projects’ website for sharing and hosting neuroimaging datasets, and can be accessed here: https://balsa.wustl.edu/study/show/v0D7.
