# Supplementary material for: Toward a “treadmill test” for cognition: Improved prediction of general cognitive ability from the task activated brain
Source: Hum Brain Mapp. 2020 May 4;41(12):3186–97. doi: 10.1002/hbm.25007 (PMC7375130; doi:10.1002/hbm.25007)
Supplement: Supplementary file 1 — Appendix S1. Supporting Information. [file HBM-41-3186-s001.docx]

**Supplement**

**Supplemental Tables**

| Task | Contrast Name | Contrast Number | File |
| --- | --- | --- | --- |
| WM | 2BK | 9 | MNINonLinear/Results/tfMRI_WM/tfMRI_WM_hp200_s2_level2_MSMAll.feat/GrayordinatesStats/cope9.feat/cope1.dtseries.nii |
| WM | 0BK | 10 | MNINonLinear/Results/tfMRI_WM/tfMRI_WM_hp200_s2_level2_MSMAll.feat/GrayordinatesStats/cope10.feat/cope1.dtseries.nii |
| WM | 2BK-0BK | 11 | MNINonLinear/Results/tfMRI_WM/tfMRI_WM_hp200_s2_level2_MSMAll.feat/GrayordinatesStats/cope11.feat/cope1.dtseries.nii |
| GAMBLING | PUNISH | 1 | MNINonLinear/Results/tfMRI_GAMBLING/tfMRI_GAMBLING_hp200_s2_level2_MSMAll.feat/GrayordinatesStats/cope1.feat/cope1.dtseries.nii |
| GAMBLING | REWARD | 2 | MNINonLinear/Results/tfMRI_GAMBLING/tfMRI_GAMBLING_hp200_s2_level2_MSMAll.feat/GrayordinatesStats/cope2.feat/cope1.dtseries.nii |
| GAMBLING | PUNISH-REWARD | 3 | MNINonLinear/Results/tfMRI_GAMBLING/tfMRI_GAMBLING_hp200_s2_level2_MSMAll.feat/GrayordinatesStats/cope3.feat/cope1.dtseries.nii |
| MOTOR | AVG | 7 | MNINonLinear/Results/tfMRI_MOTOR/tfMRI_MOTOR_hp200_s2_level2_MSMAll.feat/GrayordinatesStats/cope7.feat/cope1.dtseries.nii |
| LANGUAGE | MATH-STORY | 3 | MNINonLinear/Results/tfMRI_LANGUAGE/tfMRI_LANGUAGE_hp200_s2_level2_MSMAll.feat/GrayordinatesStats/cope3.feat/cope1.dtseries.nii |
| SOCIAL | RANDOM | 1 | MNINonLinear/Results/tfMRI_SOCIAL/tfMRI_SOCIAL_hp200_s2_level2_MSMAll.feat/GrayordinatesStats/cope1.feat/cope1.dtseries.nii |
| SOCIAL | TOM | 2 | MNINonLinear/Results/tfMRI_SOCIAL/tfMRI_SOCIAL_hp200_s2_level2_MSMAll.feat/GrayordinatesStats/cope2.feat/cope1.dtseries.nii |
| SOCIAL | TOM-RANDOM | 6 | MNINonLinear/Results/tfMRI_SOCIAL/tfMRI_SOCIAL_hp200_s2_level2_MSMAll.feat/GrayordinatesStats/cope6.feat/cope1.dtseries.nii |
| RELATIONAL | MATCH | 1 | MNINonLinear/Results/tfMRI_RELATIONAL/tfMRI_RELATIONAL_hp200_s2_level2_MSMAll.feat/GrayordinatesStats/cope1.feat/cope1.dtseries.nii |
| RELATIONAL | REL | 2 | MNINonLinear/Results/tfMRI_RELATIONAL/tfMRI_RELATIONAL_hp200_s2_level2_MSMAll.feat/GrayordinatesStats/cope2.feat/cope1.dtseries.nii |
| RELATIONAL | REL-MATCH | 4 | MNINonLinear/Results/tfMRI_RELATIONAL/tfMRI_RELATIONAL_hp200_s2_level2_MSMAll.feat/GrayordinatesStats/cope4.feat/cope1.dtseries.nii |
| EMOTION | FACES-SHAPES | 3 | MNINonLinear/Results/tfMRI_EMOTION/tfMRI_EMOTION_hp200_s2_level2_MSMAll.feat/GrayordinatesStats/cope3.feat/cope1.dtseries.nii |

Supplemental Table S1: List of filenames for the tasks and contrasts used in the study.

**Supplemental Figures**

**
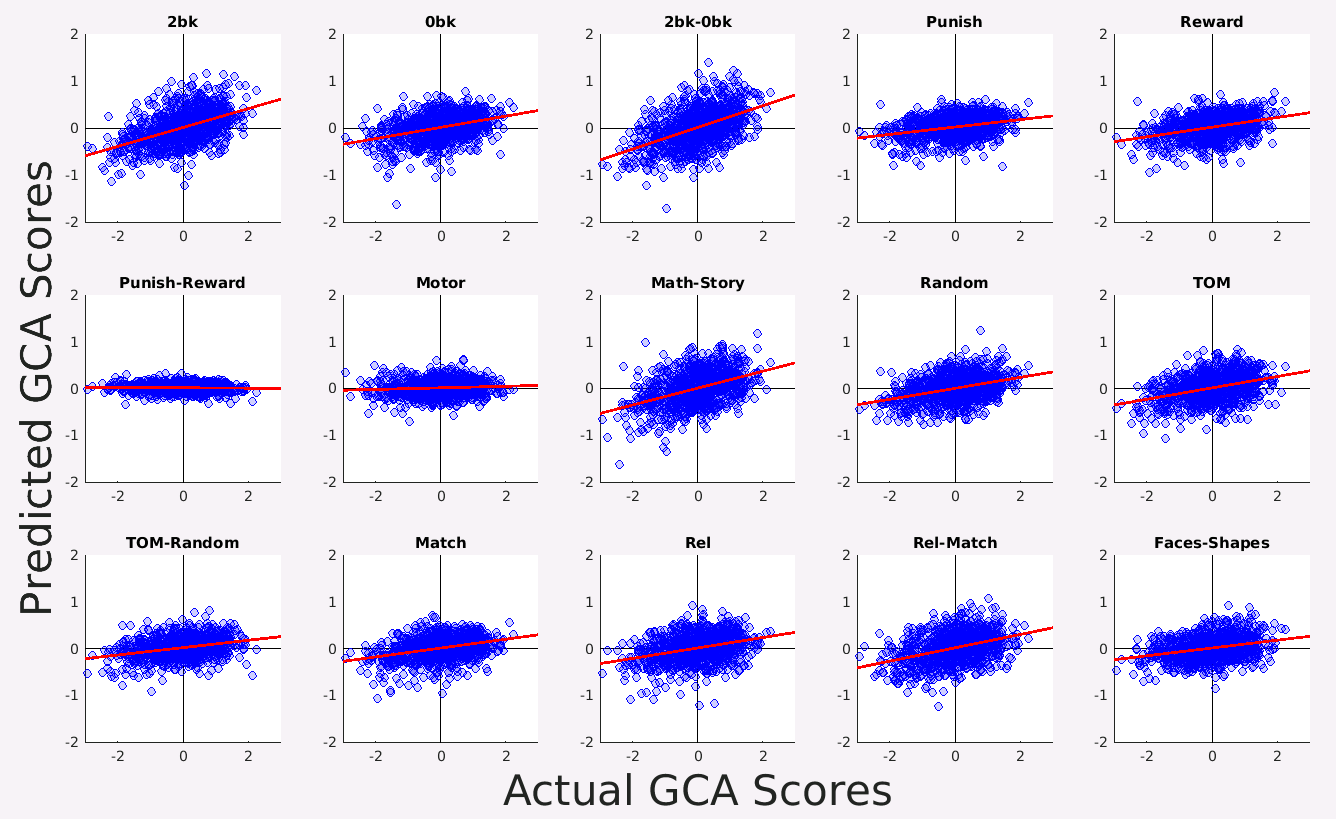
**

*Supplemental Figure S1: Scatter plots showing BBS prediction results for GCA scores for 15 task contrasts.*


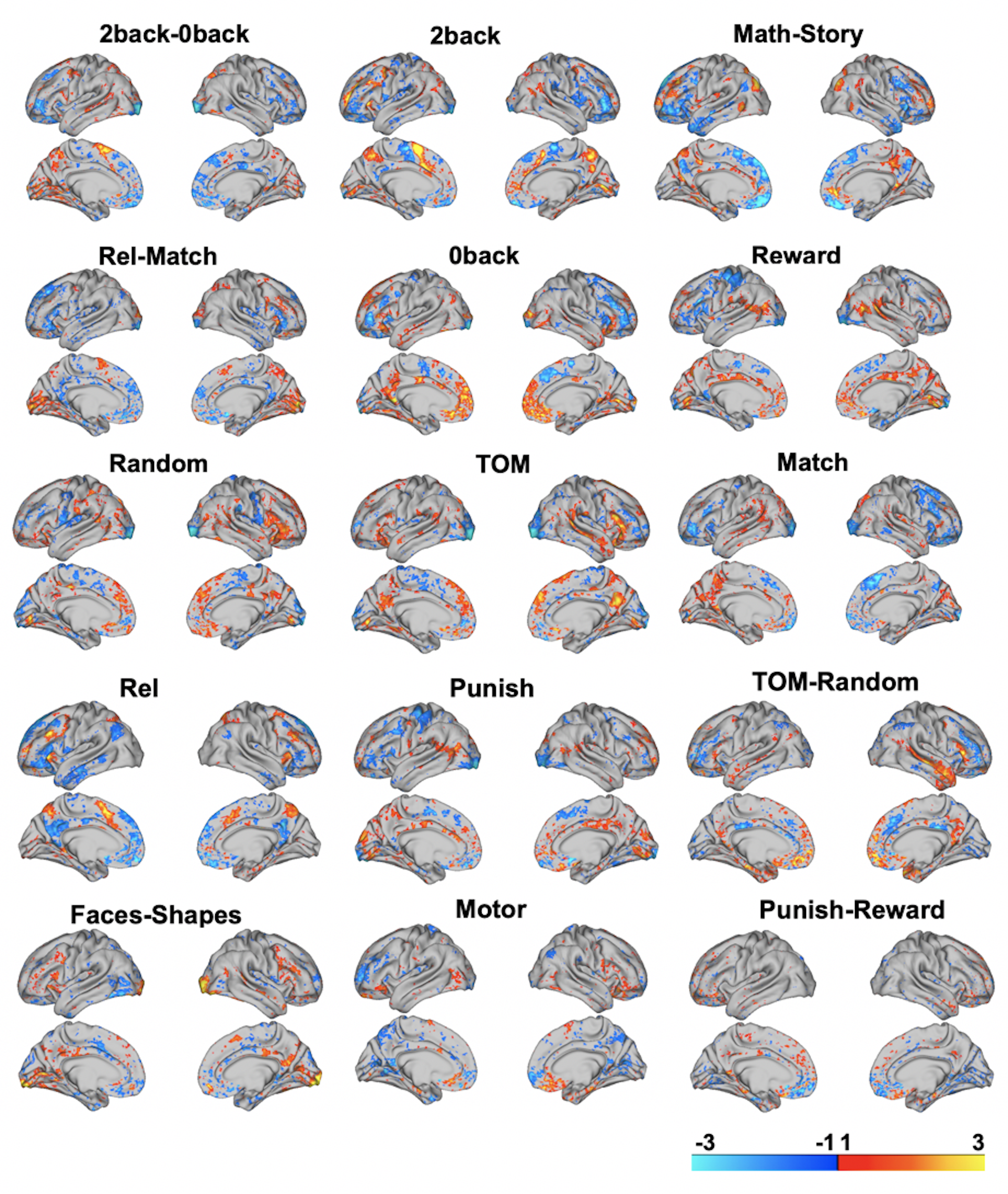


*Supplemental Figure S2: Consensus predictive maps for all 15 task contrasts. Each consensus map captures aggregate patterns across a 75-component brain basis set model for prediction of GCA scores (see Methods for details).*
